# Impact of migalastat on cerebral outcomes in fabry disease – results from the prospective observational FAMOUS trial

**DOI:** 10.1186/s42466-025-00440-w

**Published:** 2025-12-16

**Authors:** Momoko Choudhury, Malte Lenders, Pauline Laufer, Max Masthoff, Sima Canaan-Kühl, Christine Kurschat, Nicole Muschol, Julia B. Hennermann, Markus Cybulla, Jessica Kaufeld, Eva Brand, Antje Bischof

**Affiliations:** 1 Department of Neurology, University Hospital Muenster, Muenster, Germany; 2 Center of Neurology, Department of Neuroimmunology, University Hospital Bonn, Bonn, Germany; 3 Department of Internal Medicine D, and Interdisciplinary Fabry Center (IFAZ), University Hospital Muenster, Muenster, Germany; 4 Clinic of Radiology, University and University Hospital Muenster, Muenster, Germany; 5 Department of Nephrology and Medical Intensive Care, Fabry Zentrum, Center for Rare Kidney Diseases (CeRKiD), Campus Charité Mitte, Charité-Universitätsmedizin Berlin, Berlin, Germany; 6 Department II of Internal Medicine, Center for Molecular Medicine Cologne, Center for Rare Dis-eases, University of Cologne, Cologne, Germany; 7 International Center for Lysosomal disorders (ICLD), Department of Pediatrics, University Medical Center Hamburg-Eppendorf, Hamburg, Germany; 8 Department for Pediatric and Adolescent Medicine, University Medical Center Mainz, Villa Metabolica, Mainz, Germany; 9 Department of Nephrology and Rheumatology, Nephrologicum-MGL MVZ, Muellheim, Germany; 10 Department of Nephrology and Hypertension, Hannover Medical School, Hannover, Germany

**Keywords:** Fabry disease, MRI, Migalastat, Stroke, White matter hyperintensities, Cerebral small vessel disease, Basilar artery diameter, Central vein sign, Multiple sclerosis

## Abstract

**Background:**

Fabry disease (FD) is an X-linked lysosomal storage disorder caused by mutations in the α-galactosidase A (*GLA*) gene, leading to an increased risk for white matter lesion (WML), stroke and cerebral microbleeds. Utilizing MRI data from the prospective observational FAMOUS study we assessed MRI characteristics of FD and treatment effects of migalastat.

**Methods:**

19 patients with pathogenic (PV) and 14 patients with likely benign genetic variants (LBV: p.A143T, p.D313Y, and p.S126G) underwent MRI at baseline and 24 month-follow up under migalastat treatment. WML load, using Fazekas and Scheltens scores, basilar artery diameter (BAD), and the occurrence of strokes and cerebral microbleeds were assessed. Patients were compared by variant type (PV/LBV) and presence of arterial hypertension.

**Results:**

WML load was low to moderate and remained stable. Four PV patients showed progress by visual examination. WML load was similar between PV and LBV groups. Patients with arterial hypertension had a higher Scheltens score. PV patients had higher BAD. No patient showed cerebral microbleeds. One PV patient with coincident multiple sclerosis demonstrated a positive central vein sign.

**Conclusion:**

Our data suggest that microangiopathic lesion load remains relatively stable under migalastat. Antihypertensive therapy may be important to reduce WML in FD. Further studies are needed to assess the cerebral effect of migalastat therapy.

**Supplementary Information:**

The online version contains supplementary material available at 10.1186/s42466-025-00440-w.

## Background

Fabry disease (FD) is a rare X-linked lysosomal storage disorder caused by variants in the α-galactosidase A (*GLA*/AGAL) gene leading to impaired breakdown of glycosphingolipids and systemic accumulation of globotriaosylceramide (Gb_3_), particularly in vessels of affected organs [1]. Hence, FD patients may suffer from renal, cardiac, and neurological disease [1]. Typical neurological manifestations are early-onset strokes, transient ischemic attacks (TIA), and small fiber neuropathy [2]. Asymptomatic white matter lesions (WML) occur at an early stage of the disease, particularly in men [3]. It has been hypothesized that FD patients develop a cerebral vasculopathy due to endothelial accumulation of Gb_3_ [4]. which may lead to an up to 12-fold increased risk of strokes and TIA at a young age, where classical cardiovascular risk factors, heart failure and arrhythmia are still uncommon [5].

The posterior circulation appears particularly susceptible to vascular changes including dilation and tortuosity of the basilar artery [6]. Besides WML [6], cerebral microbleeds, lacunes, and enlarged perivascular spaces are commonly found in magnetic resonance imaging (MRI) at a young age in FD patients [7–9]. Most of these MRI-detectable abnormalities are unspecific and can also be found in patients with hypertensive microangiopathy, but commonly about one to three decades later [10, 11].

Since 2016, the chaperone therapy migalastat has been approved for the treatment of FD patients with migalastat-amenable variants [12–15]. In the prospective German Fabry migalastat observational multicenter (FAMOUS) study, migalastat treatment was demonstrated to be safe and to reduce cardiac left ventricular mass over 24 months [13]. However, data on the effect of migalastat on the central nervous system are still scarce.

Here, we assessed the effect of migalastat on secondary imaging measures from brain MRI including WML progression and on secondary clinical outcome parameters including occurrence of strokes and TIA from the FAMOUS dataset.

We compared brain MRI characteristics at baseline and after 24 months of migalastat treatment in patients with pathogenic genetic (PV) and likely benign (LBV) variants (p.A143T, p.D313Y, p.S126G) according to the guidelines for the interpretation of sequence variants by the American College of Medical Genetics and Genomics (ACMG) [16]. In LBV, a pathological reduction of AGAL activity or Gb_3_ accumulations has not been consistently shown [17].

## Methods

### Study design

FAMOUS was designed as a prospective open label multicenter observational study (NCT03135197; registration date: 2017-04-25; www.clinicaltrials.gov) [13, 14]. Inclusion criteria for the main study were (i) genetically confirmed FD with at least one FD-typical manifestation justifying the indication for therapy according to current guidelines [18, 19], (ii) detection of a migalastat amenable *GLA* variant [20] in patients ≥ 16 years of age, (iii) ERT (enzyme replacement therapy)-naïve or on treatment with ERT (agalsidase alfa, or agalsidase beta) for at least 6 months, (iv) an estimated glomerular filtration rate (eGFR) ≥ 30mL/min/1.73m^2^, no history of dialysis, no transplantation at least 4 weeks before initiation. Patients with a non-amenable *GLA* variant and likely benign variants were excluded from the study [13, 14, 16]. However, by amendment an additional subset of patients who commonly presents at FD centers due to neurological manifestations of unknown etiology (with p.A143T or p.D313Y) was included. Patients were included in the current MRI analysis if they completed MRI examinations at baseline before or under 4 months after initiation of migalastat treatment between 2016 and 2018 (mean − 9.7 months, SD [standard deviation] 11.4) and after 24 months of migalastat treatment (mean 22 months, SD 8.4). The last follow-up MRI was acquired in 2020.

### Study groups

**Fig. 1:**
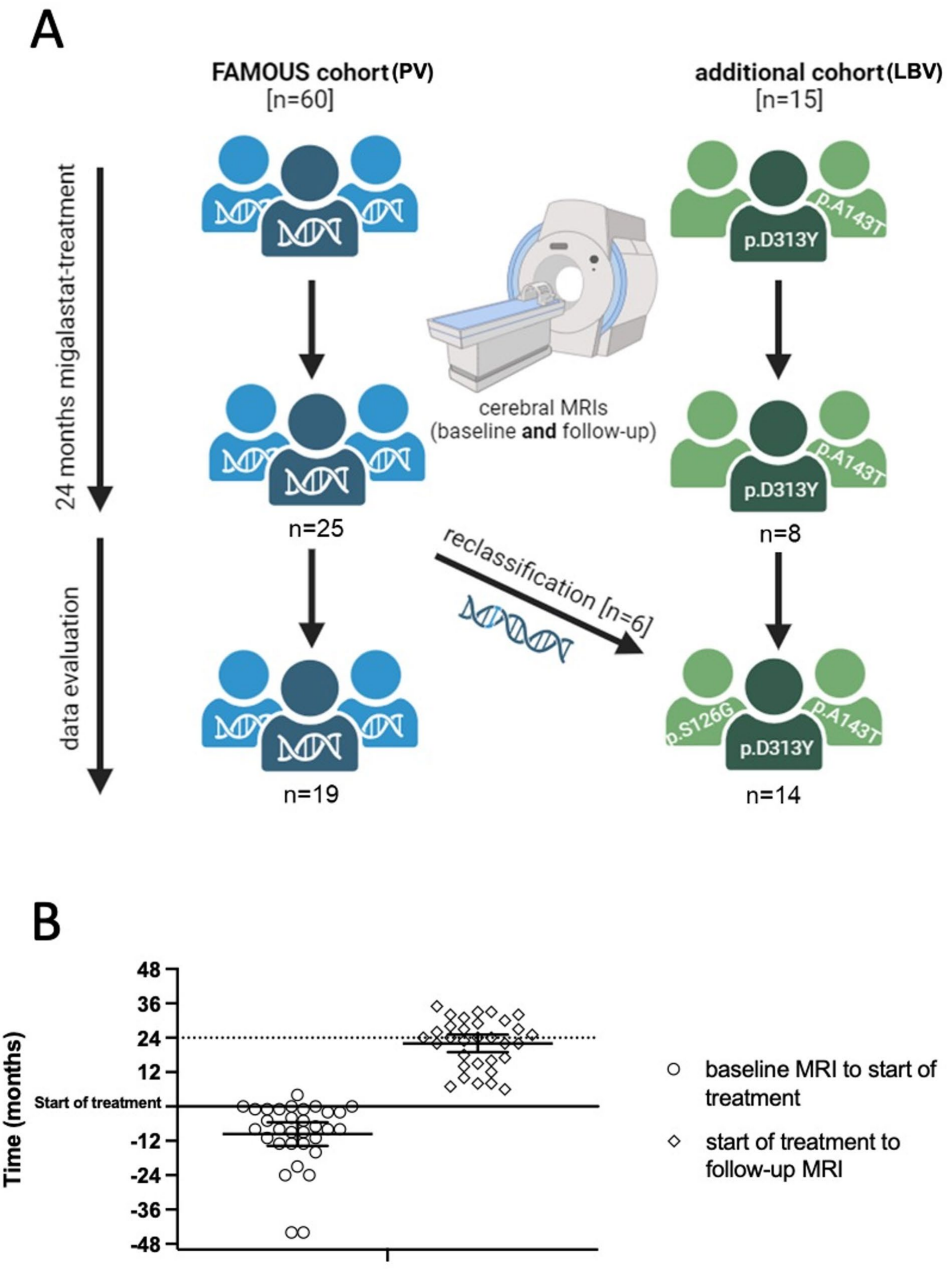
Study overview. (**A**) Overview of the study population and their analyses. MRI: magnetic resonance im-aging. PV: pathogenic variants. LBV: likely benign variants. Reclassification of α-galactosidase A variants from disease causing to likely benign was performed according to Ortiz et al. [18]. (**B**) Overview of MRI acquisition times. Baseline MRI was acquired before or up to 4 months after start of treatment (y = 0). Follow-up MRI was acquired at least 4 months after start of treatment. MRI: magnetic resonance imaging. Error bars: mean with 95% confidence interval

From the original FAMOUS cohort of 60 PV patients from 9 German Fabry centers, 25 patients from 7 German Fabry centers (Berlin, Cologne, Hamburg, Hannover, Mainz, Muellheim, Muenster) underwent MRI scans at study baseline and completed 24 month-follow-up (Fig. 1). Of these, 19 patients with PV according to ACMG (p.N34S, p.A37T, p.R118C, p.W162G, p.P205T, p.K213M, p.N215S [3x], p.M290L, p.L294S [2x], p.M296V, p.R301Q, p.N320I [4x], p.G325S) were included in the PV group for the final analysis (Supplemental Table 1) [16, 18].

From the additional cohort of patients with LBV treated with migalastat (*n* = 15), 8 patients (p.A143T [4x]; p.D313Y [4x]) underwent MRI scans at baseline and 24 month-follow-up. During the ongoing study the variant p.S126G [*n* = 6] was reclassified from pathogenic to likely benign [18]. Thus, fourteen patients with LBV were included in our final analysis (Fig. 1A). This classification is in line with recent findings by Klein et al. 2024 showing that none of these variants consistently showed reduced GLA activity or pathological Gb_3_ levels [17].

### Data assessment

Routine clinical assessment of demographic parameters, cardiovascular risk factors, FD-specific renal, cardiac and neurological parameters, and patients’ questionnaires were performed at migalastat-naïve baseline and after 24 months of treatment as previously described [13, 14]. Disease severity was assessed using the Mainz Severity Score Index (MSSI) [21].

Brain MRI was acquired using 1.5T or 3T MRI of various vendors at participating centers. The standard protocol included a T1-weighted and a T2-weighted sequence (turbo spin echo or fluid attenuated inversion recovery [FLAIR]) with a maximum of 5 mm slice thickness. Where available, diffusion-weighted, susceptibility-weighted images (SWI) and time-of-flight (ToF) MR angiography were included for analysis.

Microangiopathic pathology was classified according to Wardlaw et al. [22]. WML were scored according to Fazekas et al. and Scheltens et al. [23, 24]. We assessed enlarged perivascular spaces (EPVS) separately at basal ganglia and centrum semiovale levels as described previously [8]. EPVS were categorized from axial images using the following scale: no EPVS (0), 1–10 EPVS (1), >10 EPVS (2). Basilar artery diameter (BAD) was measured at three levels (rostral, mid, caudal) from axial T2-weighted images and averaged for statistical analysis. Stroke subtype was classified according to Doubal et al. from T2-weighted and T2-FLAIR-weighted sequences, and diffusion-weighted images, where available [25]. MRI scans were assessed by two experienced raters (MC, 3 years, AB, 15 years of subspecialty experience in neuroimaging, respectively) and blinded to the clinical data. In case of discrepant reads an independent neuroradiologist (MM) was consulted and a decision was made by consensus.

Left ventricular mass (LVM) and left ventricular mass index (LVMi) were estimated by LV cavity dimension and wall thickness at end-diastole in echocardiography (LVMi, reference range 43–95 [female] and 49–115 [male] g/m²) [26]. Presence of left ventricular hypertrophy (LVH) was defined as LVMi above reference range.

The eGFR was quantified using the Chronic Kidney Disease-Epidemiology Collaboration (CKD-EPI)-based equation based on serum creatinine (eGFRcreat) [27]. Albuminuria was defined as albumin-creatinine-ratio (ACR) >30 mg albumin per gram of creatinine from spot urine.

Antihypertensive medication included treatment with angiotensin-converting enzyme (ACE)-, angiotensin 1 receptor (AT1)-, or aldosterone receptor inhibitors, be-ta-blockers, diuretics, calcium channel antagonists, and sympatholytics.

### Data analyses

Data are presented as mean ± standard deviation (SD), median (range) or number (percentage), where appropriate. Comparison between PV and LBV patients at baseline were performed using parametric and non-parametric testing. Stepwise linear regression analysis with backward selection was used for model selection and variance inflation factor (VIF) was calculated to test for multicollinearity. Fazekas score, Scheltens score, and basilar artery diameter at baseline and follow-up were analyzed by repeated-measures-ANOVA. Age, variant type (PV vs. LBV), and diagnosis of arterial hypertension were included as covariates. *p*-values < 0.05 were considered statistically significant. Data were analyzed using IBM SPSS Statistics version 29 (IBM Corporation, Armonk, NY, USA), JMP Pro 17 and GraphPad PRISM V5.0 software (GraphPad Software, La Jolla, CA, USA). Intraclass correlation coefficients were calculated using 2-way mixed models, assuming a single rater/measurement for absolute agreement according to Shrout and Fleiss [28]. For BAD measurement (ICC = 0.973), Scheltens (ICC = 0.983) and Fazekas scores (ICC = 0.996), interrater correlations were excellent. Illustrations were created with BioRender.

## Results

### Baseline characteristics of the study groups

Twenty-five patients from 7 German Fabry centers (Berlin, Cologne, Hamburg, Hannover, Mainz, Muellheim, Muenster) underwent MRI scans at study baseline and completed 24 month-follow-up (Fig. 1). Baseline characteristics of the two groups are presented in Table 1. PV and LBV groups were similar in age and sex. As expected, PV patients had higher plasma lyso-Gb3 levels compared to LBV patients (*p* < 0.0001), increased septum thickness (*p* = 0.0056), serum NT-proBNP levels (*p* = 0.0489), and left ventricular mass (*p* = 0.008), resulting in a higher frequency of left ventricular hypertrophy (*p* = 0.027; Table 1) Noteworthy, the distribution of the main cardiac risk factors including diabetes, hypertension, and smoking showed no significant differences between both groups (Table 1). However, LBV patients tended to suffer more often from dyslipidemia (*p* = 0.057; Table 1).

**Table 1:** Baseline demographic and cardiac and renal clinical characteristics

	pathogenic variants [*n* = 19]	likely benign variants [*n* = 14]	*p*-value
females, *n*	12 (63.2)	11 (78.6)	0.4551
age at baseline, years	50 [24 to 72]	48 [29 to 63]	0.8645
age at FD diagnosis, years	47 [13 to 70 ]	46 [27 to 62]	0.9070
ERT pre-treated, *n*	14 (73.7)	9 (64.3)	0.7066
AGAL activity, nmol 4-MU/h/mg	10.8 [0.6 to 38.4]	22.4 [8.8 to 192.0]	0.0897
lyso-Gb_3_, ng/ml	3.7 [0.7 to 35.7]	0.8 [0.6 to 1.4]	0.0001
lyso-Gb_3_ below reference, *n*	3 (15.8)	14 (100.0)	0.0001
body height, cm	168 [150 to 187]	166 [156 to 188]	0.6564
body weight, kg	72.5 [54.5 to 96.0]	67.0 [46.0 to 108.0]	0.7187
BMI, kg/m²	26 [21 to 32]	26 [18 to 35]	0.7737
SBP, mmHg	123 [100 to 157]	120 [95 to 140]	0.9910
DBP, mmHg	79 [65 to 91]	80 [60 to 92]	0.9197
heart frequency, bpm	72 [54 to 81]	68 [51 to 85]	0.9052
hypertension, *n*	10 (52.6)	7 (50.0)	0.9999
anti-hypertensive treatment, *n*	11 (57.9)	7 (50.0)	0.7325
ACE-, AT1- or aldosterone inhibitor, *n*	8 (42.1)	6 (42.9)	0.9999
NSAIDs, *n*	3 (15.8)	0 (0.0)	0.2443
diabetes, *n*	1 (5.3)	1 (7.1)	0.9999
dyslipidemia, *n*	3 (15.8)	7 (50.0)	0.0569
smoking, *n*	6 (31.6)	8 (57.1)	0.1728
DS3 score, total	17 [8 to 34]	19 [13 to 23]	0.5365
MSSI score, total	19 [2 to 34]	13 [5 to 30]	0.2148
serum creatinine, mg/ml	0.70 [0.54 to 1.23]	0.77 [0.54 to 1.35]	0.9776
eGFR, ml/min/1.73 m²	101 [71 to 142]	102 [63 to 126]	0.9626
ACR, mg/g	41 [6 to 425]	30 [9 to 442]	0.5461
albuminuria,	9 (47.4)	4 (28.6)	0.1283
KTX/Dialysis, *n*	0 (0.0)	0 (0.0)	0.9999
septum thickness, mm	10 [7 to 22]	9 [6 to 11]	0.0076
LVM, g	166 [91 to 389]	120 [75 to 193]	0.0081
LVMI, g/m²	92 [57 to 198]	64 [41 to 85]	0.0024
LVH, *n*	6 (31.6)	0 (0.0)	0.0272
hs-troponin T, ng/ml	8.65 [0.03 to 33.00]	3.82 [0.01 to 18.40]	0.0883
NT-proBNP, pg/ml	142 [15 to 937]	71 [10 to 240]	0.0793
ECG abnormalities, *n*	7 (36.8)	2 (14.3)	0.2409
myocardial infarction, *n*	0 (0.0)	0 (0.0)	0.9999
arrhythmia, *n*	2 (10.5)	0 (0.0)	0.4962
pacemaker/ICD, *n*	0 (0.0)	0 (0.0)	0.9999
TIA/stroke, *n*	2 (10.5)	9 (64.3)	0.0023

ACR: albumin-creatinine ratio, AGAL: α-galactosidase A (median values of the normal control ≥ 32 nmol 4-MU/h/mg), BMI: body mass index, DBP: diastolic blood pressure, DS3: Disease Severity Score System, eGFR: estimated glomerular filtration rate (CKD-Epi-based), ERT: enzyme replacement therapy, FD: Fabry disease, ICD: implantable cardioverter device, KTX: kidney transplantation, LVM(i): left ventricular mass (index), LVH: left ventricular hypertrophy defined as LVMi > references (males: >115 g/m^2^ and females: >95 g/m^2^), lyso-Gb3: globotriaosylsphingosine with an upper limit of normal of 1.8 ng/ml, MSSI: Mainz Severity Score Index, NSAID: non-steroidal anti-inflammatory drug, SBP: systolic blood pressure, TIA: transient ischemic attack

### Effects of variant type and hypertension on cerebral microangiopathy

**Fig. 2:**
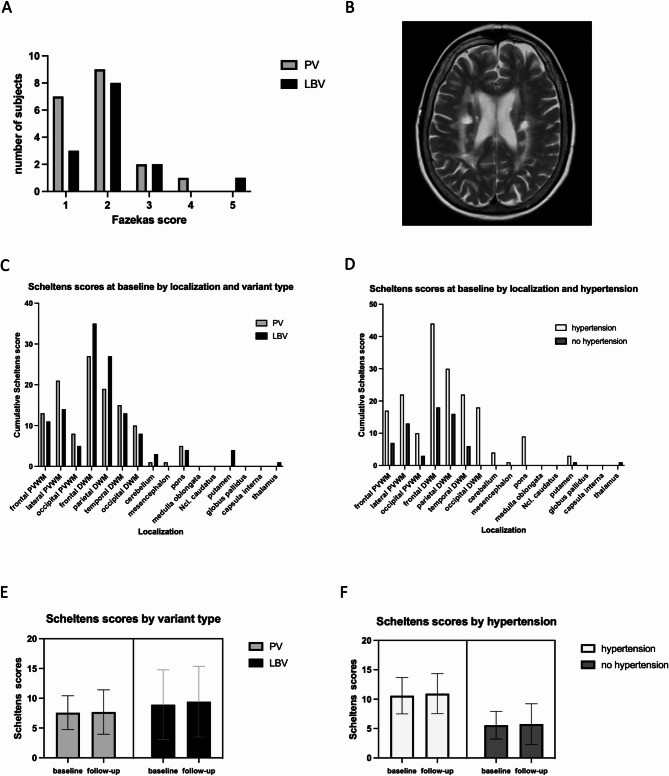
MRI analyses. (**A**) Histogram of Fazekas scores in patients with pathogenic and likely benign genetic variants. (**B**) Example of a patient with high white matter hyperintensity load. Total Fazekas score: 5. (**C**) Cumulative Scheltens scores at baseline MRI by localization and variant type. (**D**) Cumulative Scheltens scores at baseline MRI by localization and hypertension. (**E**) Mean Scheltens scores by variant type. (**F**) Mean Scheltens scores by hypertension. PV: patients with pathogenic genetic variants (light grey bars). LBV: patients with likely benign genetic variants (black bars). Patients with hypertension (white bars). Patients without hypertension (dark grey bars). PVWM: periventricular white matter. DWM: deep white matter. Ncl.: nucleus. Error bars: 95% confidence intervals. Two patients with inflammatory white matter lesions have been excluded for plots C and D

Figure 2A illustrates that WML load was low to moderate in most patients (Table 2). Figure 2B shows an example of a high white matter hyperintensity load. In both groups, WML were most frequently located in the frontal and parietal deep white matter and periventricular areas (Fig. 2C; Table 2). Occipital deep white matter and periventricular lesions were more frequent in patients with hypertension (FU: *p* = 0.03; *p* = 0.01, Fig. 2D; Table 3). Fazekas and Scheltens scores were similar in PV and LBV patients (rm-ANOVA, Fazekas: *p* = 0.270; Scheltens: *p* = 0.439) (Fig. 2E). At whole cohort level, Scheltens scores were higher in patients with arterial hypertension (rm-ANOVA, *p* = 0.043) (Fig. 2F; Table 3).

**Table 2:** MRI characteristics of patients with pathogenic and likely benign genetic variants

Parameters	pathogenic variants			likely benign variants			*p*-value*
	*N*	mean	SD	*N*	mean	SD	
BAD BL	18	3.09	0.78	13	2.49	0.65	**0.031**
BAD FU	18	2.98	0.66	13	2.44	0.61	**0.027**
Fazekas PVH BL	19	1.00	0.58	14	1.07	0.48	0.448
Fazekas DWMH BL	19	0.63	0.50	14	1.00	0.78	0.162
Fazekas total BL	19	1.63	0.76	14	2.07	1.07	0.167
Fazekas PVH FU	19	1.16	0.60	14	1.00	0.56	0.528
Fazekas DWMH FU	19	0.74	0.56	14	1.14	0.66	0.075
Fazekas total FU	19	1.89	0.94	14	2.14	1.03	0.391
Scheltens frontal PVH BL	19	0.79	0.71	14	0.79	0.70	0.988
Scheltens lateral PVH BL	19	1.26	0.45	14	1.00	0.55	0.144
Scheltens occipital PVH BL	19	0.47	0.61	14	0.36	0.63	0.598
Scheltens frontal DWMH BL	19	1.58	1.64	14	2.50	2.03	0.160
Scheltens parietal DWMH BL	19	1.11	1.29	14	1.93	2.06	0.201
Scheltens temporal DWMH BL	19	0.95	1.54	14	0.93	1.82	0.975
Scheltens occipital DWMH BL	19	0.63	1.12	14	0.57	1.09	0.878
Scheltens cerebellar ITF BL	19	0.05	0.23	14	0.21	0.80	0.409
Scheltens mesencephalic ITF BL	19	0.05	0.23	14	0.00	0.00	0.399
Scheltens pontine ITF BL	19	0.68	1.42	14	0.29	1.07	0.384
Scheltens medulla oblongata ITF BL	19	0.00	0.00^a^	14	0.00	0.00^a^	
Scheltens BGL Ncl. caudatus BL	19	0.00	0.00^a^	14	0.00	0.00^a^	
Scheltens BGL putamen BL	19	0.00	0.00	14	0.29	0.83	0.218
Scheltens BGL globus pallidus BL	19	0.00	0.00^a^	14	0.00	0.00^a^	
Scheltens BGL capsula interna BL	19	0.00	0.00^a^	14	0.00	0.00^a^	
Scheltens BGL thalamus BL	19	0.00	0.00	14	0.07	0.27	0.336
Scheltens total score BL	19	7.58	5.89	14	8.93	5.82	0.518
Scheltens frontal PVH FU	19	0.63	0.68	14	0.79	0.58	0.501
Scheltens parietal PVH FU	19	1.00	0.47	14	0.86	0.66	0.474
Scheltens occipital PVH FU	19	0.53	0.84	14	0.36	0.50	0.476
Scheltens frontal DWMH FU	19	1.79	2.02	14	3.00	1.80	0.084
Scheltens parietal DWMH FU	19	1.16	1.68	14	1.93	2.16	0.257
Scheltens temporal DWMH FU	19	0.89	1.56	14	0.71	1.27	0.725
Scheltens occipital DWMH FU	19	0.68	1.25	14	1.00	1.36	0.494
Scheltens cerebellar ITF FU	19	0.53	1.58	14	0.14	0.36	0.318
Scheltens mesencephalic ITF FU	19	0.00	0.00^a^	14	0.00	0.00^a^	
Scheltens pontine ITF FU	19	0.47	1.12	14	0.29	1.07	0.631
Scheltens medulla oblongata ITF FU	19	0.00	0.00^a^	14	0.00	0.00^a^	
Scheltens BGL Ncl. caudatus FU	19	0.00	0.00^a^	14	0.00	0.00^a^	
Scheltens BGL putamen FU	19	0.00	0.00	14	0.36	1.34	0.336
Scheltens BGL globus pallidus FU	19	0.00	0.00^a^	14	0.00	0.00^a^	
Scheltens BGL capsula interna FU	19	0.00	0.00^a^	14	0.00	0.00^a^	
Scheltens BGL thalamus FU	19	0.00	0.00^a^	14	0.00	0.00^a^	
Scheltens total score FU	19	7.68	7.73	14	9.43	5.93	0.487

^a^T cannot be calculated, *two-way, uncorrected T-test or Mann-Whitney-U-Test (significance level < 0.05, significant *p*-values are bolded)

SD: standard deviation, BAD: basilar artery diameter, BL: baseline MRI, FU: follow-up MRI, PVH: periventricular hyperintensities, DWMH: deep white matter hyperintensities, ITF: infratentorial foci of hyperintensities, BGL: basal ganglia lesions

**Table 3:** MRI characteristics of patients with and without hypertension

Parameters	no hypertension			hypertension			*p*-value*
	*N*	mean	SD	*N*	mean	SD	
BAD BL	15	2.53	0.62	16	3.13	0.82	**0.030**
BAD FU	15	2.53	0.75	16	2.96	0.55	0.082
Fazekas PVH BL	16	1.00	0.63	17	1.06	0.43	0.453
Fazekas DWMH BL	16	0.56	0.63	17	1.00	0.61	**0.037**
Fazekas total BL	16	1.56	0.89	17	2.06	0.90	**0.034**
Fazekas PVH FU	16	1.00	0.63	17	1.18	0.53	0.195
Fazekas DWMH FU	16	0.69	0.60	17	1.12	0.60	**0.042**
Fazekas total FU	16	1.69	0.87	17	2.29	0.99	**0.035**
Scheltens frontal PVH BL	16	0.56	0.63	17	1.00	0.71	0.070
Scheltens lateral PVH BL	16	1.00	0.37	17	1.29	0.59	0.094
Scheltens occipital PVH BL	16	0.25	0.45	17	0.59	0.71	0.112
Scheltens frontal DWMH BL	16	1.31	1.74	17	2.59	1.77	**0.045**
Scheltens parietal DWMH BL	16	1.13	1.54	17	1.76	1.79	0.281
Scheltens temporal DWMH BL	16	0.56	1.21	17	1.29	1.93	0.200
Scheltens occipital DWMH BL	16	0.13	0.34	17	1.06	1.34	**0.013**
Scheltens cerebellar ITF BL	16	0.00	0.00	17	0.24	0.75	0.216
Scheltens mesencephalic ITF BL	16	0.00	0.00	17	0.06	0.24	0.332
Scheltens pontine ITF BL	16	0.50	1.41	17	0.53	1.18	0.949
Scheltens medulla oblongata ITF BL	16	0.00	0.00^a^	17	0.00	0.00^a^	
Scheltens BGL Ncl. caudatus BL	16	0.00	0.00^a^	17	0.00	0.00^a^	
Scheltens BGL putamen BL	16	0.06	0.25	17	0.18	0.73	0.557
Scheltens BGL globus pallidus BL	16	0.00	0.00^a^	17	0.00	0.00^a^	
Scheltens BGL capsula interna BL	16	0.00	0.00^a^	17	0.00	0.00^a^	
Scheltens BGL thalamus BL	16	0.06	0.25	17	0.00	0.00	0.333
Scheltens total score BL	16	5.56	4.43	17	10.59	6.01	**0.011**
Scheltens frontal PVH FU	16	0.63	0.62	17	0.76	0.66	0.537
Scheltens parietal PVH FU	16	0.75	0.58	17	1.12	0.49	0.056
Scheltens occipital PVH FU	16	0.19	0.54	17	0.71	0.77	**0.033**
Scheltens frontal DWMH FU	16	1.69	1.89	17	2.88	1.96	0.085
Scheltens parietal DWMH FU	16	0.81	1.64	17	2.12	1.96	**0.048**
Scheltens temporal DWMH FU	16	0.56	1.21	17	1.06	1.60	0.325
Scheltens occipital DWMH FU	16	0.25	0.77	17	1.35	1.46	**0.011**
Scheltens cerebellar ITF FU	16	0.69	1.70	17	0.06	0.24	0.163
Scheltens mesencephalic ITF FU	16	0.00	0.00^a^	17	0.00	0.00^a^	
Scheltens pontine ITF FU	16	0.19	0.75	17	0.59	1.33	0.292
Scheltens medulla oblongata ITF FU	16	0.00	0.00^a^	17	0.00	0.00^a^	
Scheltens BGL Ncl. caudatus FU	16	0.00	0.00^a^	17	0.00	0.00^a^	
Scheltens BGL putamen FU	16	0.00	0.00	17	0.29	1.21	0.332
Scheltens BGL globus pallidus FU	16	0.00	0.00^a^	17	0.00	0.00^a^	
Scheltens BGL capsula interna FU	16	0.00	0.00^a^	17	0.00	0.00^a^	
Scheltens BGL thalamus FU	16	0.00	0.00^a^	17	0.00	0.00^a^	
Scheltens total score FU	16	5.75	6.52	17	10.94	6.61	**0.030**
^a^T can not be calculated							

*two-way, uncorrected T-test or Mann-Whitney-U-Test (significance level < 0.05, significant *p*-values are bolded)

SD: standard deviation, BAD: basilar artery diameter, BL: baseline MRI, FU: follow-up MRI, PVH: periventricular hyperintensities, DWMH: deep white matter hyperintensities, ITF: infratentorial foci of hyperintensities, BGL: basal ganglia lesions

### Progress of cerebral microangiopathy

Fazekas and Scheltens scores remained stable between baseline and follow-up (rm-ANOVA, Fazekas: *p* = 0.109; Scheltens: *p* = 0.383) (Fig. 2E, F). However, four PV patients but none of the LBV patients showed new infarctions or subtle signs of WML progression (new infarctions (*n* = 1), one new white matter lesion (*n* = 3)). The progression group showed higher Scheltens scores at baseline (*p* = 0.038) and follow-up (*p* = 0.014) compared to stable patients, but there was no difference in EPVS, BAD, infarction, sex, age, or follow-up period in months. Sensitivity analyses including the time between baseline and follow-up scans showed no significant effect of interscan interval on the results.

### EPVS, postischemic lesions and cerebral microbleeds

**Fig. 3:**
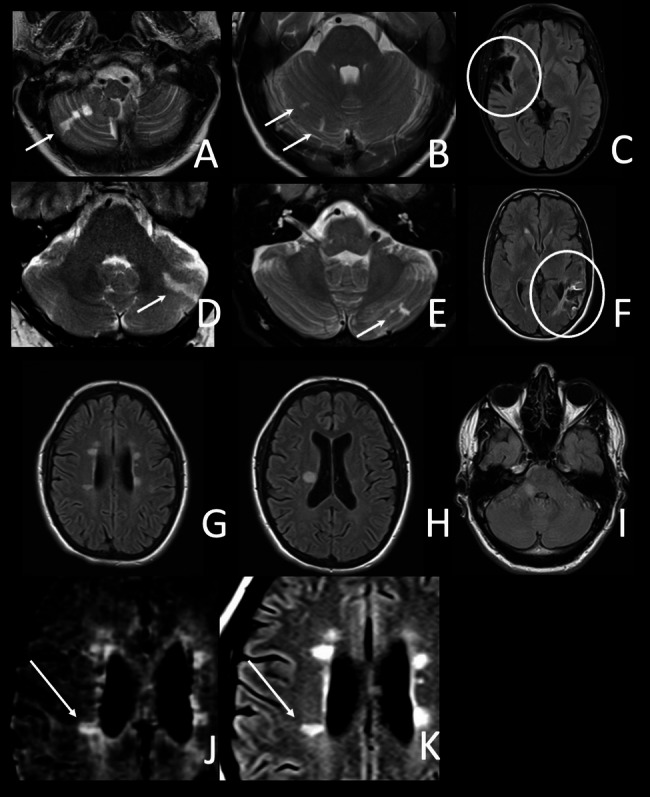
Postischemic and inflammatory lesions in MRI. (**A-F**) emboliform infarctions. A, B,D, and E show cerebellar infarctions, C and F show middle cerebral artery infarctions. (**G** and **H**) show periventricular, ovaloid lesions (Dawson’s fingers) of one patient. **I**) shows a singular lesion in the right cerebellar peduncule, (**J**) shows a central vein of a periventricular lesion on susceptibility weighted imaging (SWI). (**K**) depicts the same lesion as J on a fluid-attenuated inversion recovery (FLAIR) image. The patient shown in **G**, **H**, **J** and **K** was diagnosed with and treated for both Fabry disease and multiple sclerosis

PV and LBV patients had similar EPVS scores. Lacunes were present in four LBV patients (28.6%) and two PV patients (10.5%). Postischemic lesions on MRI were more frequently found in LBV (*n* = 4, 28.6%) than in PV patients (*n* = 2, 10.5%). Frequent infarction types were emboliform cerebellar infarction and middle cerebral artery territorial infarction (Fig. 3A-F). Interestingly, the incidence of strokes and TIA before migalastat initiation was higher in LBV patients than in PV patients (*p* = 0.0023) (Table 1). Notably, we found no cerebral microbleeds in our patients (SWI available in *n* = 23/33). One PV patient showed a postischemic lesion (right middle cerebral artery territory) with hemorrhagic transformation at baseline.

### Basilar artery diameter

While mean BAD was within physiological range in both groups (< 4.5 mm), BAD was higher in PV than LBV patients (rm-ANOVA: *p* = 0.014) and higher in patients with arterial hypertension (rm-ANOVA, *p* = 0.016) (Tables 2 and 3) [29]. Higher BAD was associated with older age (*p* = 0.037). There was no significant progress of BAD at follow-up in either group (rm-ANOVA: *p* = 0.679). BAD at baseline and follow-up was correlated with lyso-Gb_3_-levels in the whole-group analysis (Pearson’s *r* = 0.504, *p* = 0.006) and MSSI total score (BL: Pearson’s *r* = 0.380, *p* = 0.038; FU: *r* = 0.408, *p* = 0.027).

### Inflammatory white matter lesions

Two PV patients presented with WML resembling focal demyelinating lesions (Fig. 3G-K). One of them fulfilled 2017 McDonald Multiple Sclerosis (MS) criteria of spatial and temporal dissemination [30]. The respective MRI demonstrated T1-black holes and a positive central vein sign (Fig. 3J, K). Cerebrospinal fluid (CSF) analysis showed a lymphocytic pleocytosis and CSF-specific oligoclonal bands. At MS diagnosis the patient had presented with subacute onset of a right sensorimotor arm paralysis which improved after pulse steroid treatment. Another female patient presented with a large (10 mm x 8 mm) asymptomatic T2/FLAIR-hyperintense lesion within the middle cerebellar peduncle suggestive of demyelinating disease (Fig. 3I). The lesion did not show a central vein on T2* weighted fast field echo images. No lumbar puncture had been performed and 2017 McDonald criteria were not fulfilled. The lesion remained stable over 11 years and was attributed to FD.

To account for a possible bias in microangiopathic lesion load from these two atypical patients with an inflammatory phenotype of focal WMLs we performed a separate analysis of Scheltens and Fazekas scores by excluding these two subjects (*n* = 31) confirming their exclusion did not influence our results.

## Discussion

Currently, data on the cerebral effect of migalastat are scarce. Here, we present a detailed analysis of MRI data in a subset of patients from the FAMOUS trial to assess the effect of migalastat on the brain after 24 months of treatment. Our main findings are: (i) white matter lesion load remained relatively stable under treatment with migalastat; (ii) arterial hypertension is the most important driver for microangiopathic lesion load in Fabry patients treated with migalastat, similar to non-Fabry cohorts, (iii) no increased risk for cerebral microbleeds was identified in our patient subset; (iv) basilar artery diameter was within normal range, but higher in PV patients than in LBV patients, underlining this measure as a potential future imaging biomarker for treatment monitoring; (v) the central vein sign can aid diagnosis of concurrent multiple sclerosis in Fabry patients.

We found that WML load remained stable under treatment with migalastat over 24 months. On qualitative assessment three PV but no LBV patients showed one new WML under treatment with migalastat, a notion to be addressed in larger cohort studies in the future. One additional PV patient demonstrated a new middle cerebral artery territorial infarction. All four patients were female and three of them presented with cardiovascular comorbidities such as arterial hypertension, atrial fibrillation and elevated NT-proBNP. It must be considered that a new infarction or new white matter lesions could be attributed to those other risk factors not directly targeted by migalastat. LBV patients were more likely to have experienced strokes or TIAs before recruitment and to present with dyslipidemia. It can be assumed that these patients underwent genetic testing because of their cerebrovascular manifestations, leading to underestimation of other cardiovascular risk factors such as hypertension and dyslipidemia. However, these patients did not show signs of progression during the observational period. This finding may be explained by a tendency of post-stroke patients to be rigorously monitored and treated regarding cardiovascular risk factors and receive medication for secondary stroke prevention.

Arterial hypertension had a greater association with the severity of WML than the presence of a pathogenic genetic variant in our study sample. Accordingly, microangiopathic lesions were most frequently localized in the frontal and parietal deep white matter, typical for hypertensive WML [31]. Hence, microangiopathic lesions in FD may partly emerge due to hypertensive vascular stress rather than disease-specific pathophysiology [32]. This is not in line with previous investigations suggesting a minor role of other cardiovascular risk factors in the etiology of WMLs in FD [11]. Our finding may be explained by the moderate clinical affection of our cohort, but also allow the assumption that the combination of a pathogenic genetic variant and the presence of additional cardiovascular risk factors can increase the risk of white matter lesion formation. Therefore, the optimization of blood pressure and other cardiovascular risk factors may help to improve neurological out-comes in FD and should be strictly implemented in clinical practice.

To date, the impact of Fabry-specific treatments, such as migalastat and ERT, on cerebral MRI outcomes in Fabry disease has been infrequently studied. However, previous studies monitoring patients undergoing ERT have shown that the development of new white matter lesions, as well as the risk of stroke and transient ischemic attacks (TIA), were not affected by the treatment [33]. Fellgiebel et al. found relatively stable WML burden under ERT [34]. These studies chose observational periods between 6 months and 27 months, similar to our study, however, both ERT and chaperone therapy may need long-term assessment to observe radiologically detectable effects on Gb_3_ deposition.

Importantly, we did not find any cerebral microbleeds in our study sample although they have been consistently reported as a frequent finding in FD [7]. In contrast to most published FD studies our patient cohort (partly due to the presence of amenable variants) comprises predominantly patients with a moderate disease burden, including a high proportion of female patients. Our result might suggest FD patients with mild white matter pathology have a similar risk of intracerebral hemorrhage as non-FD patients. This is in line with current recommendations for consequent anti-coagulation therapy in FD patients with atrial fibrillation regardless of the CHA2DS2-VASc score [35]. Thus, direct oral anticoagulants should be considered as the first-line choice in FD patients without renal contraindications [35].

PV patients had higher BAD than LBV patients supporting previous evidence that this parameter can serve as a potential imaging biomarker to distinguish between patients with and without FD [36]. BAD has also been suggested as a biomarker for treatment monitoring in patients undergoing ERT [37]. However, in our study, both groups did not show pathologically increased BAD, and there was no correlation of BAD with lyso-Gb_3_ levels in the PV group. The small sample size and the overall low lyso-Gb_3_ levels, partially after ERT, might have influenced this result. Furthermore, arterial hypertension was a relevant risk factor for higher BAD in our sample. Vertebrobasilar dolichoectasia has been described in stroke populations, especially with posterior circulation infarctions [38]. The effect of strict cardiovascular risk factor management on BAD, however, remains to be determined. Nevertheless, our findings suggest a potential value of regular BAD screening in FD patients and the requirement of strict antihypertensive treatment.

MS is a frequent misdiagnosis in FD patients due to the inflammatory appearance of WML in some FD patients [39]. Differential diagnosis from MRI alone is currently still challenging, particularly when the cerebral lesions are localized in the periventricular and infratentorial white matter, considered typical localizations of demyelinating disease [40]. The pathogenesis of this particular lesion type in FD remains elusive. A vasculopathy including increased arterial stiffness and endothelial dysfunction resulting from cerebrovascular risk factors, particularly diabetes mellitus, has previously been reported [41, 42]. However, our case highlights that demyelinating disease can co-occur with FD [43]. The finding of WML typical of demyelinating disease should therefore prompt further investigations including CSF analysis for signs of chronic inflammation even in patients with confirmed GLA variants. Böttcher et al. reported that positive oligoclonal bands can serve as a useful biomarker in differentiating Fabry disease from inflammatory CNS disease [39]. Recently, a novel imaging biomarker, the central vein sign, has been consistently re-ported to differentiate MS lesions from other neurological diseases [44]. A single center study compared the central vein sign in a cohort of FD and MS patients demonstrating that FD patients do not show a central vein [45]. Our study demonstrated that the central vein sign can aid concurrent MS diagnosis in FD patients.

## Limitations

Due to its observational character, our study has several limitations. The acquisition of MRI data without a harmonized protocol at the various radiological centers may lead to potentially hampered WML detection and scoring. The parameters expected to be mostly affected by this heterogeneity are BAD, Fazekas and Scheltens score. Additionally, not all scans included hemoglobin-sensitive or diffusion-weighted protocols. Assessment of cerebral microbleeds was therefore performed on a smaller patient subset (*n* = 23/33). Notably, the observation period between baseline and follow-up MRI varied (Fig. 1). Although sensitivity analyses showed no effect of interscan interval variability, this remains a potential source of bias, as microangiopathic stress may develop gradually over several years. Due to the COVID-19 pandemic, some patients were unable to attend the follow-up MRI. Although the morbidity of our patient sample is comparable to that of the initial FAMOUS cohort [13, 14], a slight selection bias cannot be entirely excluded.

Due to the reclassification and thus revised treatment recommendations of the p.A143T, p.D313Y, and p.S126G variant, treatment with migalastat was stopped after the end of this study in all patients carrying p.A143T and p.D313Y. One female with p.S126G still receives migalastat due to a history of recurrent strokes (no other etiology), while two other patients with this variant were lost to follow-up.

FD is an X-linked hereditary disorder with females more mildly affected and underreported. In our study, more females than males were included (22 vs. 11), resulting in an overall lower morbidity compared to previously reported FD MRI studies. Furthermore, not all patients with disease-causing PV and none of the LBV patients presented with increased plasma lyso-Gb_3_ levels, which is considered to drive vascular pathology in FD. Because some patients were pre-treated with ERT, it can be assumed that those with PV presented with elevated plasma lyso-Gb_3_ levels at FD diagnosis.

This study reports longitudinal MRI results in PV patients treated with migalastat. Due to the observational approach of the study and the small sample size, statements about the effect of migalastat treatment on cerebral manifestations including the distinct causes of progression should be interpreted carefully but may serve as the foundation for future prospective cohort studies.

## Conclusions

In summary, our data suggest that white matter lesion load remains stable under treatment with migalastat over short term intervals. Antihypertensive treatment may be of high importance to reduce white matter lesion formation in Fabry disease. Anticoagulants and platelet inhibitors might be prescribed similarly to the general population in Fabry patients presenting with a moderate cerebral disease load and without cerebral microbleeds on baseline MRI. Further studies with larger patient cohorts, harmonized MRI protocols and longer observational periods are needed to assess the effect of migalastat therapy on cerebral outcome.

## Supplementary Information

Below is the link to the electronic supplementary material.

Supplementary Material 1

## Data Availability

All data and material are present within the main manuscript.

## Abbreviations

ACMG: American College of Medical Genetics and Genomics
ACR: Albumin-creatinine ratio
AGAL: α-galactosidase A
BAD: Basilar artery diameter
BGL: Basal ganglia lesions
BL: Baseline
BMI: Body mass index
CNS: Central nervous system
CSF: Cerebrospinal fluid
DBP: Diastolic blood pressure
DS3: Disease Severity Score System
DWM: Deep white matter
eGFR: Estimated glomerular filtration rate
EPVS: Enlarged perivascular spaces
ERT: Enzyme replacement therapy
FD: Fabry disease
FLAIR: Fluid attenuated inversion recovery
FU: Follow-up
Gb_3_: Globotriaosylceramide
GLA: α-galactosidase A gene
ICD: Implantable cardioverter device
ITF: Infratentorial foci of hyperintensities
KTX: Kidney transplantation
LBV: Likely benign genetic variants
LVH: Left ventricular hypertrophy
LVM(i): Left ventricular mass (index)
MR/MRI: Magnetic resonance imaging
MS: Multiple sclerosis
MSSI: Mainz Severity Score Index
*n*: Number
Ncl.: Nucleus
NSAID: Non-steroidal anti-inflammatory drug
NT-proBNP: N-terminal pro-B-Type natriuretic peptide
PV: Pathogenic genetic variant
PVH: Periventricular hyperintensities
PVWM: Periventricular white matter
rm-ANOVA: Repeated-measures analysis of variance
SBP: Systolic blood pressure
SD: Standard deviation
SWI: Susceptibility-weighted images
T: Tesla
TIA: Transient ischemic attack
ToF: Time of flight
VIF: Variance inflation factor
WML: White matter lesion
