# Supplementary material for: Impact of migalastat on cerebral outcomes in fabry disease – results from the prospective observational FAMOUS trial
Source: Neurol Res Pract. 2025 Dec 16;7(1):98. doi: 10.1186/s42466-025-00440-w (PMC12706964; doi:10.1186/s42466-025-00440-w)
Supplement: Supplementary file 1 — Supplementary Material 1 [file 42466_2025_440_MOESM1_ESM.docx]

| **Supplemental table 1: Overview of the identified α-galactosidase A mutations in the Fabry disease patients.** | |
| --- | --- |
| **Pathogenic variants [n=19]** | **Likely benign variants* [n=14]** |
| p.N34S (1xf), p.A37T (1xf), p.R118C (1xf), p.W162G (1xf), p.P205T (1xm), p.K213M (1xf), p.N215S (2xf; 1xm), p.M290L (1xm), p.L294S (1xf; 1xm), p.M296V (1xm), p.R301Q (1xw), p.N320I (3xf; 1xm), p.G325S (1xm) | p.S126G (5xf; 1xm), p.A143T (3xf; 1xm), p.D313Y (3xf; 1xm) |

f: females; m: male. *according to Ortiz et al. Mol Genet Metab. 2018;123:416-427
